# Colorectal cancer: towards new challenges and concepts of preventive healthcare

**DOI:** 10.3332/ecancer.2017.ed74

**Published:** 2017-11-28

**Authors:** Lidia Colace, Stefania Boccia, Ruggero De Maria, Ann Zeuner

**Affiliations:** 1 Department of Surgical Sciences, Policlinico Umberto I/Sapienza University of Rome, Viale del Policlinico, 00161 Rome, Italy; 2 Section of Hygiene, Institute of Public Health, Faculty of Medicine, Università Cattolica del Sacro Cuore, Fondazione Policlinico ‘A Gemelli’, Rome, Italy; 3 Institute of General Pathology, Università Cattolica del Sacro Cuore, Fondazione Policlinico ‘A Gemelli’, 00168 Rome, Italy; 4 Department of Oncology and Molecular Medicine, Istituto Superiore di Sanità, Viale Regina Elena 299, 00161 Rome, Italy

**Keywords:** colorectal cancer, prevention, cooperation

## Abstract

Colorectal cancer (CRC) holds the woeful record of being the most preventable but least prevented type of cancer. Although healthy lifestyles and screening programs associated to early polyp removal greatly reduce CRC incidence and CRC-related death, the overall lack of information and of effective preventive policies is promoting an overwhelming global escalation of this disease. Moreover, new challenges such as the increasing occurrence of aggressive CRC in young adults highlight underlying changes not only in the incidence but also in the nature of this disease. In this scenario, CRC prevention should also undergo a significant transformation, embracing not only individual initiatives but also issues of social and international relevance. The nascent network of countries surrounding the Mediterranean basin (COLOMED, the COLOrectal cancer MEDiterranean network) serves as a paradigm of international cooperation aimed at broadening scientific collaboration and promoting effective health policies in CRC research and prevention.

## Introduction

The global CRC burden is expected to increase by 60% to more than 2.2 million new cases and 1.1 million deaths by 2030, correlating with human development levels and with the adoption of western lifestyles [1]. Generally, low-income and middle-income countries are experiencing a sharp increase in CRC incidence and mortality, whereas industrialized countries have very high CRC rates undergoing stabilization or slight decrease. Furthermore, an unprecedented increase of CRC cases in young adults is occurring in developed countries, often presenting at advanced stages and with aggressive features [2]. Among early onset CRC patients, 30% belong to families with genetic predisposition, who require dedicated screening and management programs [3]. In the Mediterranean area, despite shared biogeographical features and nutritional habits, EU and non-EU countries present vast disparities in CRC incidence, mortality and management, as recently highlighted by the EUROMED CANCER Network survey [3]. Specifically, non-EU Mediterranean countries reported a high incidence/mortality rate, poor awareness among the population, and a lack of both comprehensive screening programs and of qualified medical personnel [4]. In this context, COLOMED is committed to promote the collaboration between Mediterranean countries in the fight against CRC. International cooperation initiatives are increasingly needed to support national actions and advocate health policies aimed at supporting CRC prevention.

## CRC prevention and screening across the Mediterranean: a synthetic overview

Since December 2003, when the Council Recommendation on CRC screening was issued, a number of countries have implemented organized screening programs. In the meantime, evidence on the efficacy and effectiveness of the fecal occult blood test (FOBT) among 50-74 year olds has been reported. A Cochrane review of randomized controlled trials showed a 25% relative risk reduction in CRC mortality (RR 0.75, CI: 0.66 - 0.84) for those attending at least one round of screening using FOBT, when adjusted for screening attendance in the individual studies [5]. Further data on effectiveness confirmed these results (27% reduction in CRC mortality in a routine screening program when restricted to participants). The European Partnership for Action Against Cancer (EPAAC) and the later Cancer Control Joint Actions (CanCon JA) called, however, for a further integration of the National Cancer Plans across EU Member States by 2020, and for the setting up of a National Structures for the governance of screening in each Member State [6]. According to a recent report of the EU Commission, the current status of CRC screening in Europe sees 23 countries with a population based program either already implemented or in the planning phase, of which 11 countries have rollout completed either nationwide or regionally [7]. Though a majority of the screening is still by FOBT, large proportions of the target population have access to screening by endoscopy (flexible sigmoidoscopy or total colonoscopy). To secure the benefits of screening, however, routine linkage between screening registries and the registries containing relevant data (morbidity and mortality for cancer) for defining the population, performance and outcome is essential and can be considered an ethical requirement of screening, as underlined in a relevant section of the European Guide on Quality Improvement in Comprehensive Cancer Control from CanCon JA [6]. Concerning Mediterranean countries outside of the EU, the WHO Country profile of 2014 [8] reports that most of them have national cancer plans, although CRC screening programs are not implemented elsewhere (e.g. Egypt, Morocco).

## Relevance of intersectoral and international initiatives in CRC prevention

It is widely accepted that CRC is closely linked to lifestyle-related factors such as nutrition, smoking, alcohol consumption and physical activity. However, such factors are not only dictated by individual choices but are closely interconnected to social health determinants such as inequalities, environment, education, employment, food, housing, water, transport and energy [9]. All these sectors will need to be addressed by appropriate actions in order to implement effective policies of CRC prevention, which will be as important as primary and secondary preventive strategies. In parallel, according to the WHO assessment that “the health of all peoples is dependent upon the fullest co-operation of individuals and States” [10], regional and global collaboration is also required to complement individual and national actions against CRC. In line with this view, in 2017 COLOMED organized an advanced theory-practice course on CRC stem cells for young scientists coming from non-EU Mediterranean countries and an international meeting where representatives from several Mediterranean countries, associations and institutions exchanged views and strategies about CRC prevention and treatment. In the future, continuous cooperation between Mediterranean countries will be crucial to support national initiatives and to promote medical and scientific collaboration.

## Conclusion

While developing countries struggle with a dramatic increase in CRC, industrialized countries deal with stable high incidence rates and changing epidemiological patterns among the population. National and international initiatives are required to successfully face this dynamic scenario and implement effective prevention strategies against CRC.
